# Effects of Musical Training in Music Therapy Following Cochlear Implantation—A Case Report

**DOI:** 10.3390/audiolres14020020

**Published:** 2024-02-22

**Authors:** Astrid Magele, Bianca Wirthner, Philipp Schoerg, Georg M. Sprinzl

**Affiliations:** 1 Department of Otorhinolaryngology, Head & Neck Surgery, University Clinic St. Poelten, Dunant-Platz 1, 3100 St. Poelten, Austria; bianca.wirthner@stpoelten.lknoe.at (B.W.); philipp.schoerg@stpoelten.lknoe.at (P.S.); georg.sprinzl@stpoelten.lknoe.at (G.M.S.); 2 Karl Landsteiner Institute of Implantable Hearing Devices, 3100 St. Poelten, Austria; 3 Department of Medicine, Karl Landsteiner Private University of Health Science Krems, 3500 Krems an der Donau, Austria

**Keywords:** cochlear implantation, music therapy, rehabilitation, music perception, quality of life

## Abstract

The most prevalent sensory impairment impacting the elderly is age-related hearing loss (HL), which affects around 65% of individuals over the age of 60 years. This bilateral, symmetrical sensorineural impairment profoundly affects auditory perception, speech discrimination, and the overall understanding of auditory signals. Influenced by diverse factors, age-related HL can substantially influence an individual’s quality of life and mental health and can lead to depression. Cochlear implantation (CI) stands as a standard intervention, yet despite advancements, music perception challenges persist, which can be addressed with individualized music therapy. This case report describes the journey of an 81-year-old musician through profound sensorineural hearing loss, cochlear implantation, and rehabilitative music therapy. Auditory evaluations, musical exercises, and quality of life assessments highlighted meaningful improvements in music perception, auditory skills, and overall satisfaction post-implantation. Music therapy facilitated emotional, functional, and musical levels of engagement, notably enhancing his ability to perceive melody, rhythm, and different instruments. Moreover, subjective assessments and audiograms indicated marked improvements in auditory differentiation, music enjoyment, and overall hearing thresholds. This comprehensive approach integrating bilateral CIs and music therapy showcased audiological and quality of life enhancements in an elderly individual with profound hearing loss, emphasizing the efficacy of this combined treatment approach.

## 1. Introduction

In the realm of geriatric health, presbycusis, or age-related hearing loss (HL), is the most common progressive sensory impairment affecting the elderly [1,2,3]. This condition can manifest as a bilateral, symmetrical sensorineural hearing loss, impacting the inner ear structures [1]. Its manifestation is characterized by difficulty in sound perception and speech discrimination, as well as altering the perception of auditory signals [4].

Age-related HL is a multifaceted condition, influenced by various factors such as genetic predisposition determining the extent of neural degeneration, pre-existing ear conditions, chronic illnesses, noise exposure, use of ototoxic medications, and lifestyle choices [1,4,5,6]. It is noteworthy that the majority, around 65% of individuals above 60 years, experience some form of disabling hearing impairment [5]. The implications of age-related HL are far-reaching, substantially affecting an individual’s quality of life and overall functioning, notably correlating with depression among older individuals [7,8]. Left unaddressed, this condition manifests as a decline in health with age and contributes substantially to years lived with disability, ranking third after depression and unintentional injuries, according to the World Health Organization (WHO) [7,9,10]. The impact of HL extends beyond personal well-being, influencing social interactions, reduced functional status, personality changes, and a high listening effort [9].

Cochlear implants (CI) have emerged as a standard intervention for hearing rehabilitation, not only for children but also for post-lingual deaf adults [11,12,13]. Technological advancements have considerably enhanced speech understanding; however, CI recipients, particularly post-lingually deaf individuals, often encounter difficulties in music perception despite improved speech comprehension [14,15,16]. This discrepancy arises from the distinct differences between electrical hearing from cochlear implants and natural acoustic hearing [17]. Both those with normal hearing and those with hearing loss depend strongly on music in their daily lives. Music is often connected to specific life events, phases, and even emotions. Music can be enjoyed on its own or in a group context for amusement, relaxation, and enjoyment. People with profound or severe hearing loss frequently miss out on the social benefits of listening to music. Recognizing the significance of music in quality of life, efforts have been directed towards approaching music perception for CI users through training and rehabilitation [18,19,20]. Music therapy (MT) has garnered attention for its positive impact on music perception in hearing-impaired individuals, showing promising results in rehabilitation programs among these listeners [14,18,21,22].

Therefore, the aim of this case report was to accompany the rehabilitation progress of an elderly (81 years old) passionate musician who encountered profound sensorineural hearing loss. It explores his journey with a new hearing situation after a bilateral cochlear implantation, aiming to enable him to pursue his life’s greatest passion: Playing and listening to music. For this purpose, audiological tests and quality of life questionnaires were utilized for evaluation, and supportive music therapy was chosen as an additional rehabilitation approach.

## 2. Materials and Methods

### 2.1. Case Presentation

The present study was conducted as part of the rehabilitative outpatient MT sessions at the Department of Otorhinolaryngology, Karl Landsteiner Private University Clinic Hospital, St.Pölten, Austria. The patient gave his written consent for the collection and publication of the data as part of a case report, and the study was conducted in accordance with the ethical standards of the clinic. An 81-year-old male presented with progressive bilateral profound sensorineural hearing loss and underwent cochlear implantation surgery. The surgical interventions, performed on separate occasions, involved the implantation of a CI (MED-EL GesmbH, Synchrony 2, Innsbruck, Tyrol, Austria) with a Flex 28 electrode. The left implantation was conducted in 2022, and the right implantation in 2021. The single unit audio processors (MED-EL GesmbH, Rondo 3, Innsbruck, Tyrol, Austria) were applied on both sides.

The cause of the deteriorating hearing is unknown. Nevertheless, the patient’s progressive hearing impairment over the last three years has considerably impacted his quality of life, especially when it comes to listening to music. In the patient’s first consultation, before the CI intervention, he passionately shared insights into his musical journey. This was the first meeting with the music therapist, Bianca Wirthner, in which he was asked about his history of hearing loss and his motivation for getting a cochlear implant. However, it should be noted that it was only possible to communicate via tablet at this meeting because the patient’s hearing loss was already so profound. It was clear that music plays an important role in his life, but he also expressed the challenges posed by his hearing impairment. Despite enduring considerable complications, particularly in regard to his musical pursuits, he persisted in playing music, driven by the tactile sensation it provides, cherishing the experience of playing various instruments such as the guitar, accordion, or piano. His determination to retain this connection highlights his high motivation to restore his hearing, primarily fueled by the desire to stage professional musical performances once more. This led to the consideration and subsequent implantation of CIs as an intervention for managing his bilateral deafness.

**Figure 1:**
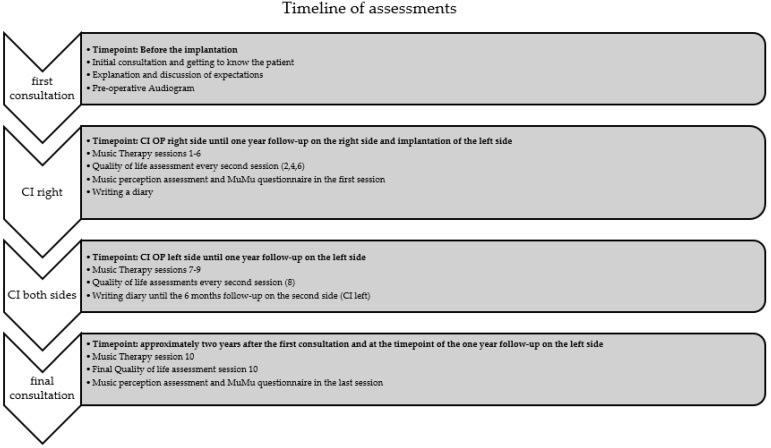
Overview of the chronological sequence of therapy sessions and assessments.

Throughout the post-implantation period, the patient has been undergoing musical rehabilitation and follow-up assessments to optimize his adaptation to the cochlear implants and to see which effect the intervention has on his auditory perception and overall communication abilities. The study data were collected at regular intervals, namely 3, 6, and 12 months for both sides after the individual implantation date. For a better overview the timeline of assessments can be seen in Figure 1.

### 2.2. Music Therapist

Mag. Bianca Wirthner, MSc, an Austrian music therapist and teacher, studied music in Graz before teaching and training students in music for elementary education. With clinical experience in geriatrics, ICU, and oncology during her music therapy studies, she has specialized in working with cochlear implant recipients since 2015 at the University Clinic St. Pölten’s ENT department. There she utilizes piano, harp, voice, and other instruments in her therapy approach.

### 2.3. Audiological Assessment

Sound field measurement, a common practice in routine audiological assessments, was conducted to ascertain hearing thresholds across multiple frequencies. These measurements were carried out in a soundproof cabin to avoid the risk of the results being biased by background noises. The degrees of hearing loss have been categorized according to the specified ranges of the American Speech–Language–Hearing Association (ASHA—“https://www.asha.org/public/hearing/degree-of-hearing-loss/ (accessed on 2 November 2023)”).

### 2.4. Music Therapy and Assessment

The subject attended ten individual MT sessions, each lasting 50 min, over a period of two years. The units were conducted in a clinic room that is not soundproof; however, the room is quiet, with minimal reverberation thanks to various sound-absorbing materials, facilitating better patient focus on sounds and music. In every setting with the CI user, the aim was to identify actual needs and to react appropriately, according to the situation, in a therapeutic way. MT works with a deliberately resource-oriented approach, and the main focus is not on disabilities or faults but on the existing resources of every individual. Therefore, each therapy section was split into the following levels:

- Level 1: Emotional level
  ○ In these therapeutic conversations, which are often the opener of each session, the CI user can mention his fears, actual needs, challenges of everyday life, etc.
- Level 2: Functional Level
  ○ Functional tasks were carried out, such as speech comprehension training, by using songs. For example, the participant had to repeat song texts from several music pieces. Practicing discrimination of voices and timbre/sounds. And explore different sounds and melodies offered (raising, descending, and constant).
- Level 3: Musical Level
  ○ Free or guided musical improvisations, experiments or/and experiences with sounds and high-quality musical instruments from all over the world, exploration of the own voice and the voices of others, singing, and playing music were just a few examples that were performed in the course of the sessions attended.

#### 2.4.1. Munich Music Questionnaire—MuMu

The MuMu questionnaire developed by MED-EL, was utilized to assess how well CI users enjoy and engage with music after their implantation surgery. This questionnaire asks about aspects such as music listening habits, sound quality perception, music recognition enjoyment levels, and engagement in music making and practice among CI users. An example of a question might be “how does music sound in general with the cochlear implant”, where the respondent can state a number between 1 and 10, where 10 means clear and 1 represents indistinct. The MuMu questionnaire consists of 25 items and includes Likert scale items, multiple-choice questions, and closed-set questions. The MuMu questionnaire serves as a valuable tool in understanding the impact of cochlear implants on individuals’ ability to enjoy and engage with music. By assessing various aspects of music perception and participation, including melody recognition, rhythm perception, and dynamic range appreciation, the MuMu questionnaire contributes to improving treatments for hearing-impaired individuals and enhancing their overall music listening experience [19].

#### 2.4.2. Music Perception

Additionally, a music perception test was performed during the first and last music therapy session. In order to be able to record a difference between direct streaming and listening to live music, all tests were carried out both with and without the “AudioLink”(AL) streaming device by MED-EL. The music perception test, based on the ‘Listen Up’ music exercises by MED-EL, included the following areas: Dynamics, pitch, tone length, timbre, melody, rhythm, and instrument recognition. Different musical pieces are played, and the participant must, for example, recognize the pitch or be able to differentiate which instrument it is. The music perception assessment consists of 7 items, and 2 to 6 points can be achieved per item. One point is scored if the respondent gets the correct answer.

### 2.5. Quality of Life Assessments

#### 2.5.1. AQoL-8D (Assessment of Quality of Life—8 Dimensions)

The AQoL-8D questionnaire is a comprehensive measure used to assess an individual’s quality of life across eight dimensions: Independent living, happiness, mental health, coping, relationships, self-worth, pain, and senses. It offers a multidimensional perspective, capturing the impact of health conditions and interventions on various aspects of an individual’s well-being. Responses to this questionnaire provide a holistic view of an individual’s quality of life with a total utility score. This score ranges from 0 to the highest score of 1, which represents the best quality of life [23].

#### 2.5.2. SSQ-B (Speech, Spatial, and Qualities of Hearing Scale—Brief)

The SSQ-B questionnaire is designed to evaluate various aspects of auditory perception and experiences in individuals with hearing impairments. It assesses speech perception, spatial hearing abilities, and the subjective qualities of hearing, including clarity, naturalness, and listening effort. This questionnaire provides insights into the functional limitations and challenges faced by individuals with hearing difficulties, understanding their auditory experiences with regard to their quality of life. Therefore, the SSQ-B questionnaire provides a scale from −5 to +5, where −5 means worse and +5 means better hearing conditions when using the device. The ability or experience is considered unchanged when it is at the midpoint of the scale (zero) [24].

## 3. Results

### 3.1. Music Therapy

As the music therapy sessions are not standardized but are always based on the individual needs of the patient, no objective results for the music therapy outcomes can be presented here. However, the music therapist has recorded observations for all sessions in order to demonstrate the therapy sessions and the patient’s progress. These impressions are now listed in abbreviated form:

- Session 1: Communication without a tablet was successful, and the patient had already practiced music at home.
- Session 2: Despite challenges in differentiating high and low tones, the therapist introduced the use of the harp and ORFF instruments, such as chimes and xylophone. Rhythm exercises proved effective, and the session explored the incorporation of digital media.
- Session 3: With improved subjective perception from daily accordion practice, the patient explored singing Wienerlieder. Successful recognition of single and multiple voices marked progress, and the therapist continued using familiar songs to reinforce the musical experience.
- Session 4: The patient demonstrated the ability to recognize words in songs, and positive life changes were reported. The therapist introduced the guitar for joint singing, highlighting advancements in both music and language perception.
- Session 5: Subjective improvement in music perception was noted, along with the patient’s daily exploration of varied music genres. The focus was set on distinguishing voices, and instrumental timbres contributed to the patient’s continued progress.
- Session 6: Following the patient’s recovery from COVID-19, new apps were introduced for challenging exercises. The patient exhibited increased engagement in musical activities without auditory support, showcasing enhanced perception of pitch, melody, and instruments.
- Session 7: Joint listening to orchestral works and the utilization of the harp for auditory exercises marked this session. Activities focused on describing music for audiological CI assessment, addressing difficulties in recognizing complex timbres.
- Session 8: The introduction of the second cochlear implant side allowed the patient to experience stereo hearing. The patient performed an unplugged concert, reporting subjective improvement in language and music perception with both CIs. Standardized exercises were successfully conducted with both systems.
- Session 9: In the three-month follow-up, the patient reported positive hearing outcomes. Daily practice resulted in improved precision in playing instruments, and the harp was introduced for challenging accompaniment.
- Session 10: Continued satisfaction with subjective music perception was reported, with daily practice involving accordion, piano, and guitar. The session addressed challenges in family life and included the successful completion of standardized exercises, showcasing sustained progress.

In addition, music perception was assessed in the sessions and thus also trained. The outcomes for the first and last sessions are shown in Table 1. The maximum score for each category is given as the number of items. Although the CI user already achieved good results in the first session, some of these could be improved by the last session.

#### 3.1.1. Diary

The patient diligently maintained a detailed diary of activities and experiences in the year following the first CI application. Notably, a substantial effort was invested in a structured training regimen, beginning with three months of standardized music training exercises, progressing to more complex musical compositions thereafter. This dedicated approach aimed to optimize the auditory and musical rehabilitation processes. The CI user held to an exact regimen of auditory training utilizing the Asklepios App for a minimum of 15 min daily, complementing these exercises with active musical engagement. To enhance their understanding of the environment and communication with others, cochlear implant patients should engage in targeted auditory exercises using the auditory training app from the start. The Asklepios App offers tasks in three difficulty levels to familiarize users with their implants. Tasks include identifying everyday sounds and interpreting numbers and “nonsense” words. Users can track their progress weekly with personalized practice statistics. To actively participate in making music, he played piano, accordion, and guitar and trained for approximately 60 min daily. This represented a strong commitment to musical involvement post-implantation.

Throughout this period, the patient reported progress in auditory differentiation abilities and a consistent and robust level of general differentiation. Subjective satisfaction ratings, reflecting the patient’s perception of the efficacy of interventions, were high across both auditory training and active musical engagement. The patient’s self-reported satisfaction scores (range 0–10) over time demonstrated a positive trend, which can be seen in Table 2.

These subjective assessments underline the patient’s perceived improvements in both auditory training and active music-making experiences across the observed periods post-implantation.

#### 3.1.2. MuMu Questionnaire

After undergoing implantation for his hearing loss, the impact on music perception and enjoyment was profound. Initially, the experience of music was altered, with a sensation that was less natural and clear compared to before. However, with the introduction of the implant, there was a return to the familiar comfort of music, scoring an 8 out of 10 in terms of music sensation and comfortable, as well as clear perception of melodies. What is remarkable is the immediate resurgence of his musical engagement post-implantation. Directly after the first fitting, he resumed a regular listening routine, experiencing a notable shift in his ability to discern rhythm and melody in the music, a skill hindered during his period of hearing loss. The breadth of his musical appreciation also saw a resurgence. Where previously limited by his hearing impairment, he now enjoys various music genres once again, spanning jazz, pop, classical, and rock. The absence of joy in music during his hearing loss phase starkly contrasts with the present, where he cherishes the pleasure of listening to diverse musical styles. His musical talents, which were challenging to carry out during the phase of hearing loss, have also reawakened. Even though he continued playing instruments, just feeling the tactile motions of the instruments, he now reported the full ability to play instruments such as the piano, accordion, and guitar. Furthermore, he also returned to singing—a joyful revival that parallels the restoration of his auditory experience after the implantation.

### 3.2. Hearing Ability

**Figure 2:**
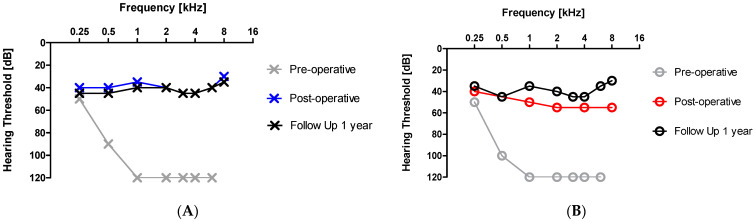
Audiogram of the left (**A**) and right (**B**) ear before and after the cochlear implant (CI) intervention.

Figure 2 shows the hearing thresholds for the left (A) and right (B) ear displayed in an audiogram. Marked in grey is the hearing ability before the CI treatment, which shows obvious deafness in the higher frequencies. After the surgery and the use of the audio processors, the hearing threshold in both ears was clearly improved. And after one year, outcomes were maintained at the same level or even improved in the case of the right ear.

### 3.3. Quality of Life Outcomes

Table 3 shows the quality-of-life results at the various control points in this study. The AQoL-8D total utility score is very high at all time points after cochlear implantation, with a value close to 1, and thus the CI user displays a high general quality of life at all time points. This is different for the quality-of-life outcomes, which are specific to hearing, speech, and the hearing solution itself. Only after the first session was an improvement, which was characterized by a positive value, observed due to the cochlear implants. After bilateral treatment, a value close to 5 in the positive range indicates a considerable improvement due to the hearing solution.

## 4. Discussion

Although it has not been defined exactly where the progressive hearing loss came from, it can be assumed that the causes are age-related, as in many older people [2,3]. In most cases, conventional hearing aids can provide assistance by amplifying the acoustic signal [25]. However, as the 81-year-old case representative could not even perceive a sound via air conduction at a hearing threshold of 120 dB, it was decided to fit Cis bilaterally. The impressive aspect of this case is not only the excellent results that were achieved during the surveys. It is also noteworthy that, due to deafness, communication was only possible using a tablet when initially getting to know each other. After the implantation, not only was it possible to have a normal conversation, but the CI user was even able to complete challenging tasks such as understanding song lyrics with ease.

Elderly individuals often decline a second bilateral implant due to potential side effects from additional surgery. Research aligns with the trend of this case report, showing that two cochlear implants (Cis) helped to enhance subjective satisfaction with hearing ability and actively making music but also improved music perception in general and the health-related quality of life when the results of the SSQ-B are considered [13,26]. While uncertainty exists about the long-term impact of music training on speech perception while listening to music [25], the CI user of this study had a better audiometric threshold, the health-related quality of life as well as music perception improved, and actively participating in challenging tasks such as playing instruments and singing was possible. This aligns with recommendations for extended training periods (more than a year) to optimize rehabilitation outcomes for those with hearing impairments [22].

The therapist’s empathetic and appreciative attitude and the developed therapeutic relationship are fundamental to the therapeutic process. The therapeutic use of music can serve as an instrument of communication, a form of expression, and a means of creativity [19,21]. Music therapy can be seen as a holistic concept of an individually oriented form of therapy. The interests of music therapy lie in flexibility within the therapeutic work between therapist and client. This fact makes it more difficult to standardize on a scientific level [15]. Nevertheless, the general approaches described in this case report can be taken as a basis and applied individually to other patients to support the rehabilitation process after CI implantation.

The patient demonstrated realistic expectations and high motivation, both of which are important characteristics for music rehabilitation [18,20]. The study’s CI user’s pre-existing passion for music underscores the importance of considering music therapy in future cases. Non-musicians can certainly still benefit from music therapy, as it offers a completely different and therefore interesting approach to supporting people with hearing loss [11,14]. However, the therapy must be adapted accordingly.

It is well recognized that acoustic and electrical hearing are considerably distinct from one another. According to earlier research, electrical hearing results in a considerably smaller dynamic range, a much steeper loudness rise, a temporal pitch that is only a few hundred Hertz, and either no tuning at all or substantially broader tuning [17]. Despite these obstacles, the MED-EL CI made it possible to hear and, for example, differentiate between different sounds in this case report. During the music therapy exercises and in the MuMu questionnaire, it was stated that the sound sensation of the tones was natural, pleasant, and clear. This was also found in the study with an older implant version of MED-EL, where a third of the CI users analyzed described the music as natural and 50% as pleasant [27].

While the study presents valuable insights into the efficacy of bilateral cochlear implants combined with music therapy for age-related hearing loss in an elderly patient, certain limitations should be considered. The single-case design restricts generalizability, and reliance on subjective measures and the absence of a control group may introduce bias. Nonetheless, the study offers a clinically relevant exploration of innovative rehabilitation approaches, employing a comprehensive assessment protocol and longitudinal follow-up to provide valuable insights into the patient’s progress over time.

In conclusion, this case report underlines the utilization of bilateral cochlear implants in combination with music therapy as a treatment for elderly people dealing with profound hearing loss, contributing valuable improvements to the audiological as well as quality of life outcomes.

## Figures and Tables

**Table 1:** Music perception training outcomes with and without the Audiolink (AL). The points indicating the number of correct answers.

Test Areas	Volume Dynamics 5 Items		Pitch 5 Items		Sound Length 5 Items		Melody 10 Items		Rhythm 5 Items		Instruments 6 Items		Timbre 2 Items	
Session/ Audiolink used	No	Yes	No	Yes	No	Yes	No	Yes	No	Yes	No	Yes	No	Yes
1	3	3	4	5	3	1	8	7	4	5	3	6	2	1
10	5	5	5	5	5	5	8	10	5	5	5	5	1	2

The first session with unilateral Cochlear implant (CI) on the right side. The last session with bilateral CI.

**Table 2:** Subjective satisfaction outcomes with hearing and active music making in the first year of using cochlear implants (CI).

Timeline Diary	1–3 Months	3–6 Months	6–12 Months
	CI Unilateral	CI Unilateral	CI Bilateral
Satisfaction hear training	7.84	8.58	8.89
Satisfaction active music making	7.14	8.82	8.67

Possible range of points reaches from 0 to 10 with 10 being the best outcome.

**Table 3:** Quality-of-life outcomes after the cochlear implantation.

Session	Status	AqoL-8D Total Utility Score	SSQ-B Overall Score
2	3 months control CI right	0.98	−1.55
4	6 months control CI right	0.99	2.80
6	12 months control CI right	0.88	0.43
8	6 months control CI left	0.95	4.24
10	12 months control CI left	0.97	3.35

## Data Availability

Data is contained within the article.
